# Complete Genome Sequencing of Thermus thermophilus Strain HC11, Isolated from Mine Geyser in Japan

**DOI:** 10.1128/MRA.00873-19

**Published:** 2019-09-26

**Authors:** Kentaro Miyazaki

**Affiliations:** a Bioproduction Research Institute, National Institute of Advanced Industrial Science and Technology (AIST), Tsukuba, Ibaraki, Japan; b Department of Computational Biology and Medical Sciences, The University of Tokyo, Kashiwa, Chiba, Japan; University of Maryland School of Medicine

## Abstract

Thermus thermophilus strain HC11 was isolated from Mine Geyser in Japan, where type strain HB8 was isolated 50 years ago. In this article, the complete genome sequence of HC11 is presented. HC11 shares the highest average nucleotide identity with HB8 among known T. thermophilus genomes (93.1%) with no genetic rearrangements.

## ANNOUNCEMENT

Thermus thermophilus is an extremely thermophilic bacterium that grows optimally between 70 and 75°C. It was first isolated by Tairo Oshima about a half-century ago from Mine Geyser in Japan (1). Since then, many *Thermus* strains have been isolated from high-temperature environments (2, 3). The strains HB8 and HB27, both isolated from Mine Geyser, have been extensively studied due to their high thermostability (4–7). Several different techniques have been used, including genetic engineering (8), protein engineering (6, 9), structural genomics (10–12), and functional genomics (13).

I collected boiling water samples at Mine Geyser on the Izu Peninsula. The sample was spread over LB (1% [wt/vol] tryptone, 0.5% [wt/vol] yeast extract, 0.5% [wt/vol] NaCl) agar (1.5% [wt/vol]) plates and incubated at 65°C overnight. Several single colonies were isolated, and DNA sequencing analysis of 16S rRNA genes suggested that all the isolates belonged to T. thermophilus (>99% identity to the T. thermophilus HB8 gene). The whole-genome sequence was analyzed in one of the strains, HC11. Cells were grown to saturation in LB broth, and genomic DNA was purified using the MagAttract high-molecular-weight (HMW) DNA kit (Qiagen). Long- and short-read sequencing was performed using GridION (Oxford Nanopore Technologies [ONT]) and MiSeq (Illumina), respectively. Software analyses throughout this study were conducted using default parameter settings.

For long-read sequencing, genomic DNA was treated with Short Read Eliminator (Circulomics). A library was constructed with 1.0 μg of the resulting DNA using a ligation sequencing kit (ONT). The library was then analyzed on a FLO-MIN 106 R9.41 flow cell (ONT) for 12 h. Base-calling was conducted using Guppy v.3.0.3 to generate 68,638 reads (956 Mb) with an average length of 13,929 bases. The raw reads were filtered (average Phred quality values of >8.0) using NanoFilt v.2.3.0 (14). The longest read was 192,749 bases.

For short-read sequencing, a Nextera DNA Flex library prep kit (Illumina) was used to generate paired-end libraries with insertions that were approximately 350 bp long. Sequencing was performed using a MiSeq reagent kit v.2 (300 cycles) with reads that were 156 bp long. Adapter sequences and low-quality data were trimmed using fastp v.0.14.1 (15), yielding 2.59 million paired-end reads, spanning 398 Mb with an average length of 153.6 bp.

**FIG 1:**
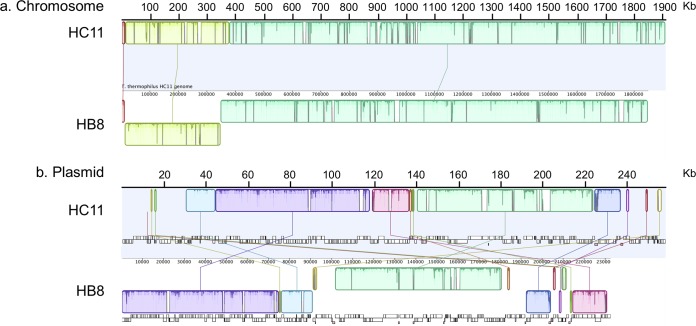
Genomic sequence alignment of T. thermophilus strains HC11 and HB8. (a) Chromosomes; (b) plasmids. The alignment was generated using Mauve software (http://darlinglab.org/mauve/mauve.html) with default settings.

The long-read and short-read data were assembled *de novo* using Unicycler v.0.4.7 (16), followed by assembly polishing with Pilon v.1.23 (17). This yielded a single circular chromosome (1,910,731 bp, G+C content of 69.4%) and a plasmid (258,759 bp, G+C content of 69.1%). The obtained sequence data were submitted to a Web-based annotation pipeline, DFAST v.1.1.0 (18), for automated annotation. The chromosome sequence of HC11 was most similar to HB8 (GenBank accession number NC_006461) among known T. thermophilus genomes, sharing an average nucleotide identity of 93.1% with HB8. Despite the known dynamic evolution patterns in *Thermus* species genomes (19, 20), no genetic rearrangements were observed (Fig. 1a). However, even though the nucleotide identity between the HC11 plasmid (pHC11) and the HB8 plasmid (pTT27) was high (97.8%), numerous genetic rearrangements were observed (Fig. 1b).

T. thermophilus strains are polyploids with four to five sets of chromosomes (21). A previous study of chromosome and plasmid copy numbers showed that they are detected in equal numbers. In the present study, the relative chromosome and plasmid copy numbers were estimated from the coverage of short reads to the complete chromosome/plasmid sequences. Sequence coverage was 180.4× ± 27.5× for the chromosome and 146.3× ± 79.8× for the plasmid. This suggested that the copy numbers were similar but a bit higher for the chromosome.

### Data availability.

The complete genome sequence of T. thermophilus HC11 is available from DDBJ/EMBL/GenBank under the accession numbers AP019801 for the chromosome and AP019802 for the plasmid. Raw sequencing data were deposited in the SRA database under the accession numbers DRR184352 (Illumina) and DRR184353 (Nanopore). The BioProject number is PRJDB8536, and the BioSample number is SAMD00178645.

## References

[1] Oshima T, Imahori K 1974 Description of *Thermus thermophilus* (Yoshida and Oshima) comb. nov., a nonsporulating thermophilic bacterium from a Japanese thermal spa. Int J Sys Evol Microbiol 24:102–112. doi:10.1099/00207713-24-1-102.

[2] Lyon PF, Beffa T, Blanc M, Auling G, Aragno M 2000 Isolation and characterization of highly thermophilic xylanolytic *Thermus thermophilus* strains from hot composts. Can J Microbiol 46:1029–1035. doi:10.1139/w00-075. 11109491

[3] Miyazaki K, Tomariguchi N 2019 Complete genome sequences of *Thermus thermophilus* strains AA2-20 and AA2-29, isolated from Arima Onsen in Japan. Microbiol Resour Announc 8:e00820-19. doi:10.1128/MRA.00820-19. 31371550 PMC6675998

[4] Nagahari K, Koshikawa T, Sakaguchi K 1980 Cloning and expression of the leucine gene from *Thermus thermophilus* in *Escherichia coli*. Gene 10:137–145. doi:10.1016/0378-1119(80)90131-6. 6993286

[5] Yamada T, Akutsu N, Miyazaki K, Kakinuma K, Yoshida M, Oshima T 1990 Purification, catalytic properties, and thermal stability of threo-Ds-3-isopropylmalate dehydrogenase coded by *leuB* gene from an extreme thermophile, *Thermus thermophilus* strain HB8. J Biochem 108:449–456. doi:10.1093/oxfordjournals.jbchem.a123220. 2277037

[6] Miyazaki K, Yaoi T, Oshima T 1994 Expression, purification, and substrate specificity of isocitrate dehydrogenase from *Thermus thermophilus* HB8. Eur J Biochem 221:899–903. doi:10.1111/j.1432-1033.1994.tb18805.x. 8181473

[7] Lopez-Lopez O, Cerdan ME, Gonzalez-Siso MI 2015 *Thermus thermophilus* as a source of thermostable lipolytic enzymes. Microorganisms 3:792–808. doi:10.3390/microorganisms3040792. 27682117 PMC5023265

[8] Koyama Y, Hoshino T, Tomizuka N, Furukawa K 1986 Genetic transformation of the extreme thermophile *Thermus thermophilus* and of other *Thermus* spp. J Bacteriol 166:338–340. doi:10.1128/jb.166.1.338-340.1986. 3957870 PMC214599

[9] Yaoi T, Miyazaki K, Oshima T, Komukai Y, Go M 1996 Conversion of the coenzyme specificity of isocitrate dehydrogenase by module replacement. J Biochem 119:1014–1018. doi:10.1093/oxfordjournals.jbchem.a021316. 8797105

[10] Yokoyama S, Hirota H, Kigawa T, Yabuki T, Shirouzu M, Terada T, Ito Y, Matsuo Y, Kuroda Y, Nishimura Y, Kyogoku Y, Miki K, Masui R, Kuramitsu S 2000 Structural genomics projects in Japan. Nat Struct Biol 7:943. doi:10.1038/80712. 11103994

[11] Iino H, Naitow H, Nakamura Y, Nakagawa N, Agari Y, Kanagawa M, Ebihara A, Shinkai A, Sugahara M, Miyano M, Kamiya N, Yokoyama S, Hirotsu K, Kuramitsu S 2008 Crystallization screening test for the whole-cell project on *Thermus thermophilus* HB8. Acta Crystallogr F Struct Biol Cryst Commun 64:487–491. doi:10.1107/S1744309108013572. PMC2496871 18540056

[12] Cava F, Hidalgo A, Berenguer J 2009 *Thermus thermophilus* as biological model. Extremophiles 13:213–231. doi:10.1007/s00792-009-0226-6. 19156357

[13] Kim K, Okanishi H, Masui R, Harada A, Ueyama N, Kuramitsu S 2012 Whole-cell proteome reference maps of an extreme thermophile, *Thermus thermophilus* HB8. Proteomics 12:3063–3068. doi:10.1002/pmic.201100375. 22887638

[14] De Coster W, D’Hert S, Schultz DT, Cruts M, Van Broeckhoven C 2018 NanoPack: visualizing and processing long-read sequencing data. Bioinformatics 34:2666–2669. doi:10.1093/bioinformatics/bty149. 29547981 PMC6061794

[15] Chen S, Zhou Y, Chen Y, Gu J 2018 fastp: an ultra-fast all-in-one FASTQ preprocessor. Bioinformatics 34:i884–i890. doi:10.1093/bioinformatics/bty560. 30423086 PMC6129281

[16] Wick RR, Judd LM, Gorrie CL, Holt KE 2017 Unicycler: resolving bacterial genome assemblies from short and long sequencing reads. PLoS Comput Biol 13:e1005595. doi:10.1371/journal.pcbi.1005595. 28594827 PMC5481147

[17] Walker BJ, Abeel T, Shea T, Priest M, Abouelliel A, Sakthikumar S, Cuomo CA, Zeng Q, Wortman J, Young SK, Earl AM 2014 Pilon: an integrated tool for comprehensive microbial variant detection and genome assembly improvement. PLoS One 9:e112963. doi:10.1371/journal.pone.0112963. 25409509 PMC4237348

[18] Tanizawa Y, Fujisawa T, Kaminuma E, Nakamura Y, Arita M 2016 DFAST and DAGA: Web-based integrated genome annotation tools and resources. Biosci Microbiota Food Health 35:173–184. doi:10.12938/bmfh.16-003. 27867804 PMC5107635

[19] Kumwenda B, Litthauer D, Reva O 2014 Analysis of genomic rearrangements, horizontal gene transfer and role of plasmids in the evolution of industrial important *Thermus* species. BMC Genomics 15:813. doi:10.1186/1471-2164-15-813. 25257245 PMC4180962

[20] Tripathi C, Mishra H, Khurana H, Dwivedi V, Kamra K, Negi RK, Lal R 2017 Complete genome analysis of *Thermus parvatiensis* and comparative genomics of *Thermus* spp. provide insights into genetic variability and evolution of natural competence as strategic survival attributes. Front Microbiol 8:1410. doi:10.3389/fmicb.2017.01410. 28798737 PMC5529391

[21] Ohtani N, Tomita M, Itaya M 2010 An extreme thermophile, *Thermus thermophilus*, is a polyploid bacterium. J Bacteriol 192:5499–5505. doi:10.1128/JB.00662-10. 20729360 PMC2950507

